# Diabetes mellitus is associated with altered Alzheimer's disease pathology

**DOI:** 10.1002/alz.72002

**Published:** 2026-11-10

**Authors:** Min Wu, Ying Luan, Xiaowen Xu, Xiaoxie Mao, Huiying Lin, Kun He, Qi Huang, Jie Zhang, Sheng Kuang, Yihui Guan, for the Alzheimer's Disease Neuroimaging Initiative, the Health and Aging Brain Study (HABS‐HD) Team, Sid O'Bryant, Binyin Li, Qihao Guo, Fang Xie, Benyan Luo

**Affiliations:** ^1^ Department of Neurology, First Affiliated Hospital, School of Medicine Zhejiang University Hangzhou China; ^2^ Department of Nuclear Medicine & PET Center Huashan Hospital Fudan University Shanghai China; ^3^ Department of Radiology Zhongda Hospital School of Medicine Southeast University Nanjing China; ^4^ Department of Radiology Tongji Hospital School of Medicine Tongji University Shanghai China; ^5^ Center for Rehabilitation Medicine Rehabilitation and Sports Medicine Research Institute of Zhejiang Province Hangzhou China; ^6^ Department of Rehabilitation Medicine Zhejiang Provincial People's Hospital Hangzhou Medical College Hangzhou China; ^7^ The D‐Lab Department of Precision Medicine GROW ‐ Research Institute of Oncology and Reproduction Maastricht University Maastricht the Netherlands; ^8^ Institute for Translational Research University of North Texas Health Science Center at Fort Worth Fort Worth USA; ^9^ Department of Neurology and Institute of Neurology Ruijin Hospital Shanghai Jiao Tong University School of Medicine Shanghai China; ^10^ Department of Gerontology Shanghai Sixth People's Hospital Shanghai Jiao Tong University School of Medicine Shanghai China; ^11^ MOE Frontiers Center for Brain Science Fudan University Shanghai China

**Keywords:** Alzheimer's disease, amyloid beta, dementia, diabetes mellitus, tau accumulation

## Abstract

**INTRODUCTION:**

Diabetes mellitus (DM) is a major risk factor for dementia, yet it remains unclear whether DM accelerates cognitive decline through amyloid beta (Aβ) or tau accumulation in Alzheimer's disease (AD).

**METHODS:**

In this study, we included 2784 participants from three independent observational cohorts: the Chinese Preclinical Alzheimer's Disease Study (CPAS), the Alzheimer's Disease Neuroimaging Initiative (ADNI), and Health and Aging Brain Study–Health Disparities (HABS‐HD). We examined the associations of DM with Aβ positron emission tomography (PET), tau PET, plasma phosphorylated tau (p‐tau)217, and longitudinal cognitive change. We also tested whether DM modified the associations of Aβ and plasma p‐tau217 with subsequent tau accumulation and explored the association of metformin use with tau accumulation.

**RESULTS:**

We found that DM was associated with lower global Aβ burden and lower tau accumulation in CPAS and ADNI, in which cognitive impairment was more common than in HABS‐HD. The associations of Aβ and plasma p‐tau217 with tau accumulation were weaker among individuals with DM. In addition, DM was associated with reduced propagation of tau from early to later stage Braak regions. Longitudinal analyses in ADNI indicated that individuals with DM exhibited slower tau‐related cognitive decline. In exploratory analyses, metformin use among individuals with DM was associated with lower tau accumulation compared to individuals without DM.

**DISCUSSION:**

These findings suggest that DM is associated with an attenuation of amyloid‐ and tau‐related processes, particularly in participants with cognitive impairment, and further suggest that diabetes‐related mechanisms and its management may modulate the progression of AD pathology.

## Highlights

- Diabetes mellitus was associated with lower global amyloid beta (Aβ) burden and slower tau accumulation.
- Associations of Aβ and plasma phosphorylated tau 217 with tau accumulation were weaker among individuals with diabetes mellitus.
- Diabetes mellitus was associated with reduced tau propagation from early to later Braak regions.
- Individuals with diabetes mellitus showed slower tau‐related cognitive decline.

## 1 INTRODUCTION

Diabetes mellitus (DM) affects approximately one in ten adults with prevalence projected to rise to 1.31 billion by 2050.^1^ DM is a risk factor for cognitive decline and dementia.^2^, ^3^, ^4^ Large epidemiologic studies consistently demonstrate that DM nearly doubles the risk of dementia ^5^, ^6^, ^7^. However, the effect of DM on cognitive trajectories was inconsistent, while most investigations have assumed DM accelerates Alzheimer's disease (AD) pathology and cognitive decline. Some longitudinal studies indicate that DM is associated with an increased risk of progression to AD and with faster cognitive decline ^8^, ^9^, whereas others have observed DM is linked to slower or unchanged rate of cognitive decline among patients with established AD ^10^, ^11^. This heterogeneity raises a question of how DM contributes to dementia and whether it acts through AD‐related neuropathology.

AD is characterized by the accumulation of amyloid beta (Aβ) plaques and the aggregation and spread of pathological tau neurofibrillary tangles ^12^, ^13^, ^14^. The extent to which DM modifies these core AD pathologies remains controversial. Some studies report greater Aβ burden on positron emission tomography (PET) in individuals with DM ^15^, while others find no clear association ^16^, ^17^. Tau pathology has been examined less extensively, and findings are controversial. While earlier studies reported higher cerebrospinal fluid (CSF) total and phosphorylated tau (p‐tau) in DM ^16^, recent multicenter data indicate that DM is associated with lower CSF p‐tau, with notable heterogeneity between population‐based and memory clinic cohorts ^18^. Taken together, existing evidence challenges the notion that DM uniformly exacerbates typical AD pathology.

Blood biomarkers, particularly p‐tau217, have emerged as cost‐effective, easily accessible, and highly sensitive tools to detect and monitor AD pathology ^19^, ^20^. Plasma p‐tau217 shows high accuracy for identifying abnormal brain Aβ and tau burdens even in a preclinical stage and is thought to be related to both the Aβ and tau pathology ^21^, ^22^. However, it remains unclear whether plasma p‐tau217 differs by DM status and whether DM modifies the relationships of Aβ burden and plasma biomarkers with tau pathology and cognitive decline.

To address these gaps, we examined Aβ and tau PET alongside plasma tau biomarkers in participants from three independent multicenter cohorts in 2784 participants with both Aβ and tau PET scans. We compared Aβ and tau burden, and plasma p‐tau217 levels between individuals with and without diabetes. We then tested whether DM modifies (1) the association between plasma p‐tau217 levels and tau accumulation, (2) the association between Aβ burden and tau accumulation, and (3) the spatial propagation of tau from early stage regions to later stage Braak regions. Finally, we evaluated whether DM alters the trajectory of tau‐associated longitudinal cognitive decline.

## 2 METHODS

### 2.1 Participants

**RESEARCH IN CONTEXT**

1. **Systematic review**: We searched PubMed for studies examining diabetes in relation to cognitive decline, amyloid beta (Aβ), tau, or Alzheimer's disease (AD) biomarkers. Epidemiological evidence consistently links diabetes to dementia risk, whereas autopsy, fluid biomarker, and positron emission tomography (PET) studies have reported absent, inconsistent, or inverse associations with AD pathology. Few studies have jointly evaluated longitudinal amyloid PET, tau PET, plasma phosphorylated tau (p‐tau)217, regional tau propagation, and cognition across independent cohorts.
2. **Interpretation**: We found that diabetes was associated with lower Aβ burden, slower tau accumulation and propagation, attenuation of Aβ– and p‐tau217–related tau accumulation, and slower tau‐related cognitive decline, particularly in cognitively impaired participants. Exploratory analyses reported that metformin use among individuals with diabetes mellitus (DM) was associated with lower tau accumulation compared to individuals without DM.
3. **Future directions**: Prospective studies should determine whether these associations reflect diabetes biology, treatment, or competing vascular pathology, while incorporating diabetes duration, glycemic control, vascular burden, medication exposure, and repeated AD biomarkers.

A total of 2784 participants were included from three independent cohorts: the Chinese Preclinical Alzheimer's Disease Study (CPAS),^23^ Alzheimer's Disease Neuroimaging Initiative (ADNI), and the Health and Aging Brain Study–Health Disparities (HABS‐HD). Study protocols were approved by the institutional review boards of all participating institutions and written informed consent was obtained from all participants.

CPAS is an ongoing prospective, multicenter study initiated in 2019 to characterize multimodal biomarkers of AD in a Chinese population, with recruitment from memory clinics at three hospitals in Shanghai ^23^. Detailed inclusion and exclusion criteria for this cohort have been described previously ^24^, ^25^. ADNI is a longitudinal, multicenter study launched in 2003 across North America to track AD progression. HABS‐HD is a community‐based longitudinal study designed to investigate biomarkers of AD and related dementias in diverse older adults.

In CPAS, dementia was diagnosed according to the 2018 National Institute on Aging–Alzheimer's Association (NIA‐AA) criteria and mild cognitive impairment (MCI) was defined using a modified Jak–Bondi neuropsychological test across three cognitive domains, that is, episodic memory, language function, and executive function ^26^, ^27^. In ADNI, diagnoses followed established ADNI Clinical Core criteria ^28^, and in HABS‐HD diagnoses were made by decision‐tree algorithm followed by expert consensus review.^29^ Participants with MCI or dementia were grouped as cognitively impaired (CI); all others without cognitive impairment were classified as cognitively unimpaired (CU).

In CPAS and HABS‐HD, DM was identified based on medical history obtained through structured interviews. In ADNI, DM status was determined through medical history databases and medication records (documented use of insulin or hypoglycemics). Participants were therefore classified as DM or non‐DM. Eligibility criteria included a documented diagnosis of DM or confirmed absence of DM, availability of both Aβ and tau PET scans acquired within 6 months of each other, and availability of 3T magnetic resonance imaging (MRI).

### 2.2 Plasma p‐tau 217

Plasma samples were collected from participants concurrently with PET scan (within 6 months). Plasma p‐tau217 was available in a subset of participants across cohorts. In CPAS, plasma p‐tau217 was measured using the commercial ALZpath p‐tau217 immunoassay (ALZpath, Inc). ^20^, ^30^ In ADNI, plasma p‐tau217 was measured using the same ALZpath assay on the Quanterix Simoa HD‐X platform at the Quanterix Accelerator Laboratory, with protocols available through the ADNI database. In HABS‐HD, plasma p‐tau217 was measured on the Quanterix Simoa platform.

### 2.3 Neuroimaging

In CPAS, Aβ and tau PET were conducted on a PET/computed tomography scanner (Biograph‐mCT Flow, Siemens). Aβ PET used ^18^F‐florbetapir (FBP, ≈ 10 mCi) with a 20‐minute emission acquisition from 50 to 70 minutes post‐injection. Tau PET used ^18^F‐MK‐6240 (185 MBq ± 10%) with a 20‐minute emission acquisition from 90 to 110 minutes post‐injection. High‐resolution T1‐weighted structural MRI was acquired on a 3T Siemens Prisma scanner using a magnetization‐prepared rapid gradient‐echo (MPRAGE) sequence (repetition time = 3000 ms; echo time = 2.56 ms; flip angle = 7°; matrix = 320 × 320; voxel size = 0.8 mm isotropic; 208 sagittal slices). PET images were reconstructed using filtered back‐projection with corrections for attenuation, normalization, scatter, and random coincidences, resulting in a final voxel size of 2.04 × 2.04 × 1.50 mm^3^.

In ADNI, Aβ PET used ^18^F‐florbetapir (≈ 10 mCi), with emission data collected as four 5‐minute frames from 50 to 70 minutes post‐injection. Tau PET used ^18^F‐flortaucipir (FTP, ≈ 10 mCi), with emission data collected as six 5‐minute frames from 75 to 105 minutes post‐injection. Structural MRI was acquired on 3T Siemens, GE, or Philips scanners using MPRAGE sequences. Detailed acquisition and preprocessing procedures are available at http://adni.loni.usc.edu/methods/pet‐analysis‐method/.

In HABS‐HD, Aβ PET was acquired using ^18^F‐florbetaben (FBB, ≈ 8.1 mCi), with a 20‐minute acquisition from 90 to 110 minutes post‐injection. Tau PET used ^18^F‐PI‐2620 (≈ 10 mCi), with a 30‐minute acquisition from 45 to 75 minutes post‐injection. Structural MRI was acquired on 3T Siemens Prisma or GE Discovery scanners using MPRAGE sequences.

### 2.4 Image processing

For CPAS and HABS‐HD, PET image preprocessing was performed using SPM12 (Wellcome Trust Centre for Neuroimaging, https://www.fil.ion.ucl.ac.uk/spm). PET images were co‐registered to each participant's T1‐weighted MRI, which were segmented into gray matter, white matter, and CSF. Spatial normalization to standard Montreal Neurological Institute (MNI) space was performed using deformation fields derived from T1‐weighted segmentation and subsequently applied to the co‐registered PET images.

For Aβ PET, regional uptake values were extracted using Centiloid standard mask images (voi_ cxt_2 mm.nii and voi_WhlCb_2 mm.nii) provided by the Global Alzheimer's Association Interactive Network (GAAIN). Standardized uptake value ratios (SUVRs) were calculated using whole cerebellum as the reference region and were conversed to the Centiloid value using tracer‐specific calibration equations (more details are available in our previous publication^27^). For tau PET, regional tau deposition was quantified for three composite regions corresponding to Braak stages I to II, III to IV, and V to VI using the Desikan–Killiany atlas. SUVRs were calculated using the inferior cerebellar gray matter as the reference region.

For ADNI, PET and MRI preprocessing and quantification followed the standardized pipelines as described by the ADNI PET Core and MRI Core, including motion correction, co‐registration to MRI, normalization, and reference region–based SUVR calculation. Amyloid PET SUVRs were expressed on the Centiloid scale, and tau PET SUVRs were derived using established ADNI reference regions. Detailed procedures are available on the ADNI website (http://ida.loni.usc.edu).

### 2.5 Statistics

All statistical analyses were conducted using R (version 4.3.1) and MATLAB (R2023b). Normality of continuous variables was assessed using the Shapiro–Wilk test. Plasma biomarker concentrations were natural log transformed. Continuous variables are reported as mean ± standard deviation (SD), and categorical variables as counts and percentages. Participants’ characteristics were compared between groups stratified by cognitive status (i.e., CU and CI) and DM status using chi‐squared tests for categorical measures and analysis of variance for continuous measures.

Associations between DM and cross‐sectional measures (Aβ PET Centiloid, tau PET SUVR, plasma p‐tau217, and Mini‐Mental State Examination [MMSE]) were examined using linear mixed‐effects models. All models were adjusted for age, sex, education, cognitive status, and apolipoprotein E (*APOE*) ε4 carrier status. To account for multiple comparisons, false discovery rate (FDR) correction was applied.

The modification effect of DM was evaluated by including interactions (DM × plasma p‐tau217 and DM × Aβ PET Centiloid) in models predicting tau PET SUVR. To assess whether DM modified the association between early and later tau deposition, additional models were fitted in which tau accumulation in later Braak‐stage regions (Braak III–IV or Braak V–VI SUVR) was modeled as a function of the interaction between early‐stage tau accumulation (Braak I–II SUVR) and DM status.

Longitudinal analyses were conducted in the ADNI cohort. Individual MMSE slopes were estimated using linear regression over time. Linear mixed‐effects models were fitted to evaluate whether baseline tau PET SUVR, in interaction with DM status, predicted longitudinal change in MMSE, adjusting for the same covariates. To further characterize the relationships among DM, AD pathology, and longitudinal cognition change, structural equation modeling was performed using the lavaan package in R.

Finally, exploratory effects of antidiabetic treatments on AD pathology were evaluated using multivariable linear regression models adjusted for age, sex, education, cognitive status, and *APOE* ε4 carrier status, with post hoc pairwise comparisons between medication groups. All statistical tests were two sided, with a *p* value < 0.05 considered statistically significant.

## 3 RESULTS

### 3.1 Demographics

This study included 2784 participants from the CPAS (*n* = 734), ADNI (*n* = 835), and the HABS‐HD (*n* = 1215), all of whom underwent both Aβ PET and tau PET imaging (Table 1). Participants in ADNI were older than those in CPAS and HABS‐HD (mean [SD] age, 73.7 [7.9] vs. 68.1 [8.2] and 65.7 [8.5] years). The proportion of CI participants varied across cohorts, ranging from 27.9% in HABS‐HD to 44.7% in ADNI and 66.3% in CPAS. CI participants had lower education attainment, a higher prevalence of *APOE* ε4 carriers, and a greater proportion of Aβ positivity compared to CU participants. Female participants accounted for 453 (61.7%) in CPAS, 430 (51.5%) in ADNI, and 735 (60.5%) in HABS‐HD; diabetes prevalence was 15.7% in CPAS, 13.1% in ADNI, and 27.3% in HABS‐HD. Longitudinal cognitive assessments were available for a subset of 625 participants in the ADNI cohort over a mean (SD) follow‐up duration of 3.5 (1.7) years. Detailed baseline and longitudinal characteristics are reported in Table 1 and Table S1 in supporting information.

**TABLE 1:** Demographic information.

	CPAS (*N* = 734)			ADNI (*N* = 835)			HABS‐HD (*N* = 1215)			
Characteristic	CU (*n* = 247)	CI (*n* = 487)	*p* value	CU (*n* = 462)	CI (*n* = 373)	*p* value	CU (*n* = 876)	CI (*n* = 339)	*p* value	Total (*N* = 2784)
Age, mean (SD), years	65.4 (8.2)	69.4 (7.9)	<0.001	72.7 (7.5)	74.9 (8.2)	<0.001	65.4 (8.3)	66.3 (9.2)	.13	68.7 (8.9)
Sex, no. (%)			.37			<0.001			<0.001	
Female	158 (64.0)	295 (60.6)		272 (58.9)	158 (42.4)		571 (65.2)	164 (48.4)		1618 (58.1)
Male	89 (36.0)	192 (39.4)		190 (41.1)	215 (57.6)		305 (34.8)	175 (51.6)		1166 (41.9)
Education, mean (SD), years	13.1 (3.3)	10.8 (3.9)	<0.001	16.7 (2.3)	16.0 (2.6)	<0.001	14.5 (3.4)	13.4 (3.5)	<0.001	14.2 (3.8)
*APOE* ε4 carriers, no. (%)	52 (21.1)	227 (46.6)	<0.001	149 (32.3)	166 (44.5)	<0.001	250 (28.5)	121 (35.7)	.02	965 (34.7)
Amyloid‐beta positive, no. (%)	46 (18.6)	324 (66.5)	<0.001	137 (29.7)	204 (54.7)	<0.001	133 (15.2)	89 (26.3)	<0.001	933 (33.5)
Diabetes (DM), no. (%)	40 (16.2)	75 (15.4)	.78	56 (12.1)	53 (14.2)	.37	221 (25.2)	111 (32.7)	.01	556 (20.0)
Baseline biomarkers										
Plasma p‐tau217, mean (SD), pg/mL	0.4 (0.2)	0.9 (0.6)	<0.001	0.4 (0.3)	0.6 (0.7)	.01	0.4 (0.3)	0.6 (0.6)	<0.001	0.6 (0.5)
Plasma GFAP, mean (SD), pg/mL	56.5 (33.3)	122.5 (98.0)	<0.001	179.1 (87.8)	188.8 (100.7)	.47	117.6 (66.1)	113.8 (73.3)	.61	124.4 (87.6)
Amyloid PET, mean (SD), Centiloids	2.6 (17.6)	36.0 (38.3)	<0.001	19.5 (33.5)	46.9 (51.8)	<0.001	8.1 (22.3)	16.1 (31.2)	<0.001	16.5 (39.4)
Tau PET (Braak I–II), mean (SD), SUVR	0.9 (0.2)	1.3 (0.4)	<0.001	1.1 (0.1)	1.3 (0.3)	<0.001	1.3 (0.1)	1.4 (0.2)	<0.001	1.2 (0.3)
Tau PET (Braak III–IV), mean (SD), SUVR	1.0 (0.1)	1.5 (0.7)	<0.001	1.1 (0.1)	1.2 (0.3)	<0.001	1.1 (0.1)	1.2 (0.2)	<0.001	1.2 (0.4)
Tau PET (Braak V–VI), mean (SD), SUVR	0.9 (0.1)	1.3 (0.6)	<0.001	1.0 (0.1)	1.1 (0.2)	<0.001	1.0 (0.1)	1.1 (0.1)	<0.001	1.1 (0.3)
MMSE score, mean (SD)	28.1 (1.6)	22.1 (5.5)	<0.001	29.1 (1.2)	26.6 (3.4)	<0.001	28.4 (1.7)	26.3 (3.3)	<0.001	26.9 (3.9)
Longitudinal data										
Participants with follow‐up, no. (%)	/	/	/	349 (75.5)	276 (74.0)	.61	/	/	/	/
Follow‐up duration, mean (SD), years	/	/	/	3.9 (1.6)	2.9 (1.7)	<0.001	/	/	/	/

Abbreviations: ADNI, Alzheimer's Disease Neuroimaging Initiative; *APOE*, apolipoprotein E; CI, cognitively impaired; CPAS, Chinese Preclinical Alzheimer's Disease Study; CU, cognitively unimpaired; DM, diabetes mellitus; GFAP, glial fibrillary acidic protein; HABS‐HD, Health and Aging Brain Study–Health Disparities; MMSE, Mini‐Mental State Examination; PET, positron emission tomography; p‐tau, phosphorylated tau; SD, standard deviation; SUVR, standardized uptake value ratio.

### 3.2 Lower Aβ and tau accumulation in DM

We first compared Aβ and tau accumulation between participants with and without DM within each cohort. In CPAS and ADNI, participants with DM exhibited lower global Aβ burden and lower tau accumulation in temporoparietal regions (Figure 1A–B). After adjustment for age, sex, education, cognitive status, and *APOE* ε4 carrier status, diabetes was associated with lower Aβ Centiloids in CPAS (standardized β = −0.10; 95% confidence interval [95% CI], –0.17 to –0.04; *p* = 0.002, FDR corrected) and ADNI (β = −0.08; 95% CI, −0.14 to −0.03; *p* = 0.013, FDR corrected). DM was also associated with lower tau accumulation across Braak regions in both cohorts (all *p* < 0.05, FDR corrected). In HABS‐HD, no significant difference between DM and non‐DM was observed in either Aβ burden (β = −0.04; 95% CI, −0.09 to 0.01; *p* = 0.32) or tau accumulation (Braak I–II: β = 0.02; 95% CI, −0.04 to 0.07; *p* = 0.489; Braak III–IV: β = 0.05; 95% CI, −0.01 to 0.10; *p* = 0.277; Braak V–VI: β = 0.06; 95% CI, 0.01 to 0.12; *p* = 0.194, FDR corrected).

**FIGURE 1:**
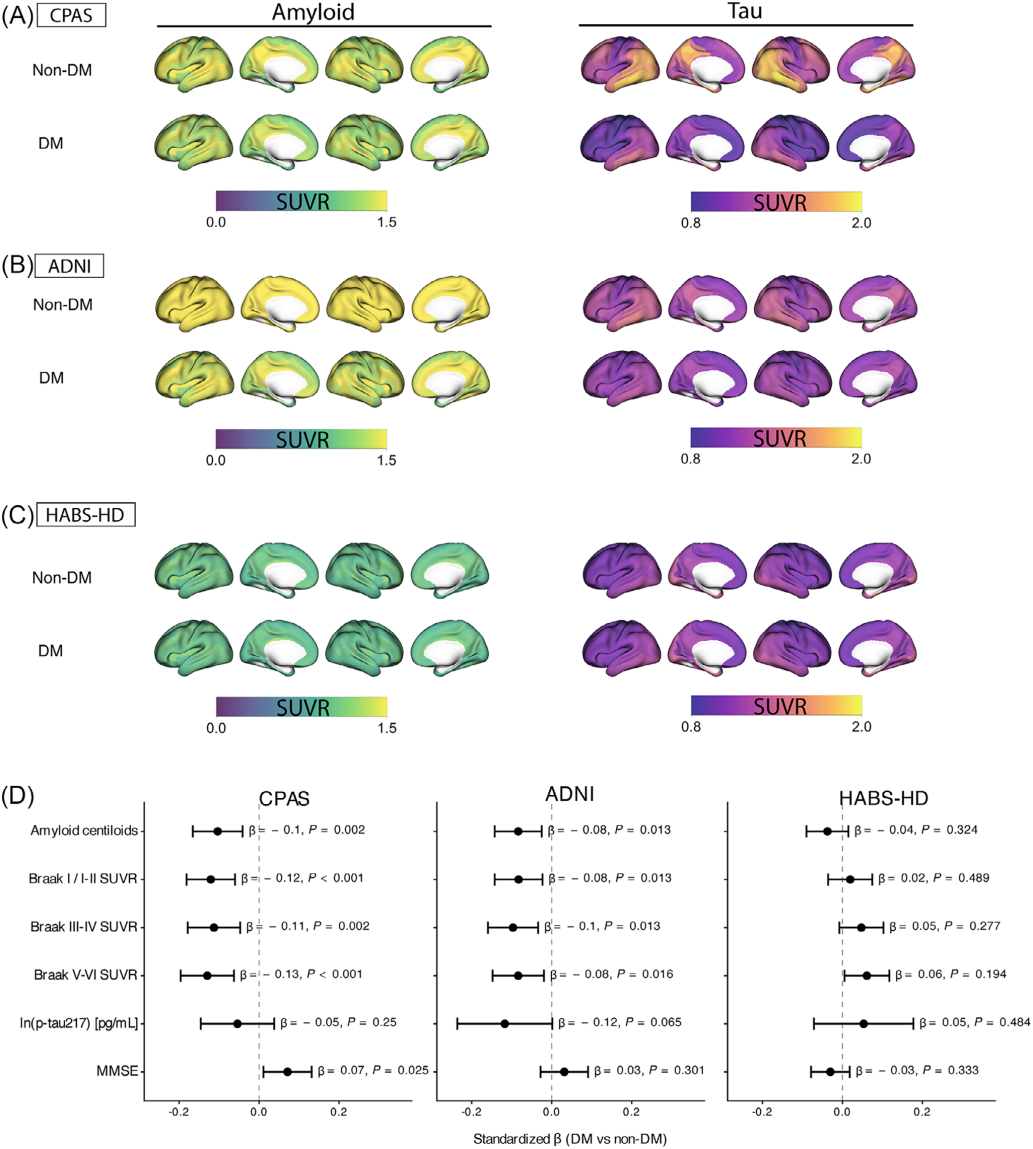
Association of diabetes mellitus with amyloid and tau burden. A–C, Cortical surface maps display the spatial distribution of amyloid (left) and tau (right) burden in individuals with and without DM across the CPAS (A), ADNI (B), and HABS‐HD (C) cohorts. D–F, Forest plots show the association of DM status (DM vs. non‐DM) with amyloid burden, tau accumulation, plasma p‐tau217, and MMSE score. Effect sizes are presented as standardized regression coefficients (β); negative values indicate lower amyloid or tau burden, or lower MMSE scores in the DM group compared to the non‐DM group. Error bars denote 95% CIs. All models were adjusted for age, sex, education, cognitive status, and *APOE* ε4 carrier status. *p* values were corrected for multiple comparisons. ADNI, Alzheimer's Disease Neuroimaging Initiative; *APOE*, apolipoprotein E; CI, confidence interval; CPAS, Chinese Preclinical Alzheimer's Disease Study; DM, diabetes mellitus; HABS‐HD, Health and Aging Brain Study–Health Disparities; MMSE, Mini‐Mental State Examination.

When stratified by cognitive status, that is, CU and CI, the associations of diabetes with lower Aβ and tau accumulation were primarily observed in CI participants in CPAS and ADNI (Figure S1 in supporting information). These associations remained consistent after additional adjustment for baseline MMSE scores. Among CU participants, associations were weaker and heterogeneous across cohorts: diabetes was associated with higher late‐stage tau in HABS‐HD, whereas associations were not consistently observed in CPAS or ADNI (Figure S1). Additional analyses stratified by Aβ status and analyses with further adjustment for white matter hyperintensity volume are presented in Figures S6 and S7 in supporting information, respectively. The associations of *APOE* ε4 carrier status with AD biomarkers within the DM and non‐DM groups are shown in Figure S8 in supporting information.

### 3.3 Diabetes attenuated plasma p‐tau217– and Aβ–associated tau accumulation

We examined whether the association between plasma p‐tau217 and tau accumulation differed according to DM status. In linear mixed‐effects models adjusted for age, sex, education, *APOE* ε4 status, and cognitive status, higher plasma p‐tau217 was associated with higher tau accumulation across Braak regions in all three cohorts. However, this association was attenuated in participants with DM, as shown by significant DM × p‐tau217 interactions in CPAS (Braak I–II: standardized β = −0.24, 95% CI −0.42 to −0.05, *p* = 0.012; Braak III–IV: β = −0.26, 95% CI −0.46 to −0.07, *p* = 0.007; Braak V–VI: β = −0.32, 95% CI −0.53 to −0.12, *p* = 0.002; Figure 2A) and ADNI (Braak I: β = −0.34, 95% CI −0.64 to −0.05, *p* = 0.024; Braak III–IV: β = −0.47, 95% CI −0.77 to −0.16, *p* = 0.003; Braak V–VI: β = −0.47, 95% CI −0.78 to −0.16, *p* = 0.003; Figure 2B). In HABS‐HD, DM attenuated the association between p‐tau217 and tau PET SUVR in moderate‐ and late‐stage Braak regions (Braak III–IV: β = −0.53, 95% CI −0.82 to −0.24, *p* < 0.001; Braak V–VI: β = −0.56, 95% CI −0.86 to −0.26, *p* < 0.001), but not in Braak I and II (β = −0.14, 95% CI −0.44 to 0.17, *p* = 0.38; Figure 2C).

**FIGURE 2:**
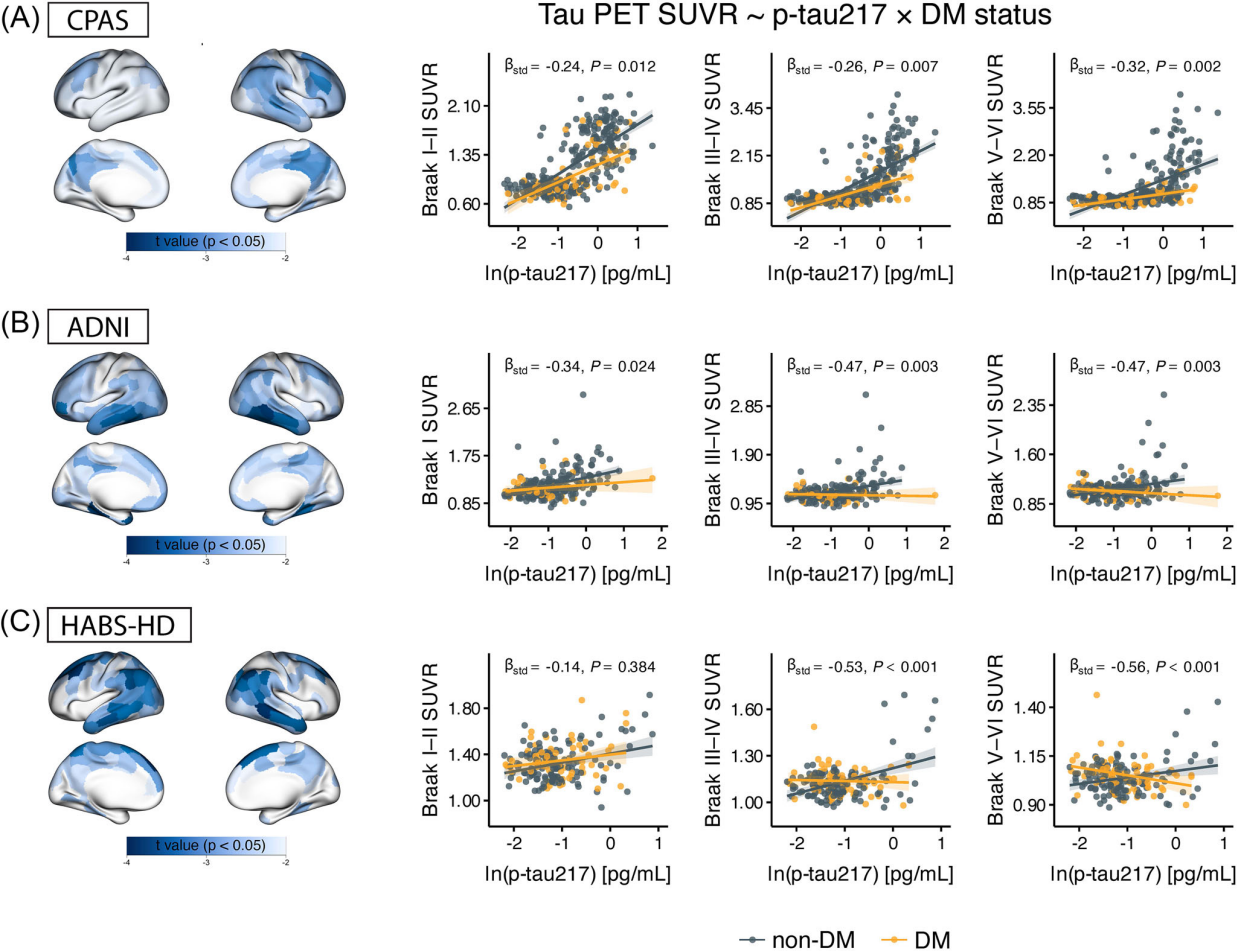
Interaction between diabetes mellitus status and plasma p‐tau217 on tau accumulation. A–C, Cortical surface maps show *t* values for the interaction between DM status and plasma p‐tau217 on regional tau accumulation across the CPAS (A), ADNI (B), and HABS‐HD (C) cohorts. Blue regions (negative *t* values) indicate areas where DM is associated with attenuated p‐tau217–related tau accumulation. D–F, Scatter plots illustrate the effect of DM status on the association between plasma p‐tau217 levels and regional tau accumulation across Braak regions (I–VI). Each point represents an individual participant. Solid lines show fitted linear regression models and shaded areas indicate 95% CIs. β values correspond to the interaction coefficients derived from regression models adjusted for age, sex, education, cognitive status, and *APOE* ε4 carrier status. ADNI, Alzheimer's Disease Neuroimaging Initiative; *APOE*, apolipoprotein E; CI, confidence interval; CPAS, Chinese Preclinical Alzheimer's Disease Study; DM, diabetes mellitus; HABS‐HD, Health and Aging Brain Study–Health Disparities; p‐tau, phosphorylated tau.

We next tested whether DM similarly modified the association between Aβ burden and tau accumulation. DM significantly attenuated the Aβ–tau association in late Braak stages in CPAS (Braak V–VI: β = −0.26, 95% CI −0.44 to −0.07, *p* = 0.006; Figure 3A) and in moderate Braak regions in ADNI (Braak III–IV: β = −0.22, 95% CI −0.43 to −0.01, *p* = 0.039; Figure 3B). No significant modification of the Aβ–tau association by DM was detected in HABS‐HD at any Braak stage (all *p* > 0.05; Figure 3C). Stratified analyses by CU and CI indicated that these attenuating effects of DM on p‐tau217–tau and Aβ–tau associations were primarily observed among CI participants (Figures S2–S3 in supporting information). In comparison, DM did not modify the glial fibrillary acidic protein (GFAP)‐related Aβ and tau burden as indicated by no significant interactions between DM and Aβ or tau accumulation (all *p* > 0.05, Figure S4 in supporting information).

**FIGURE 3:**
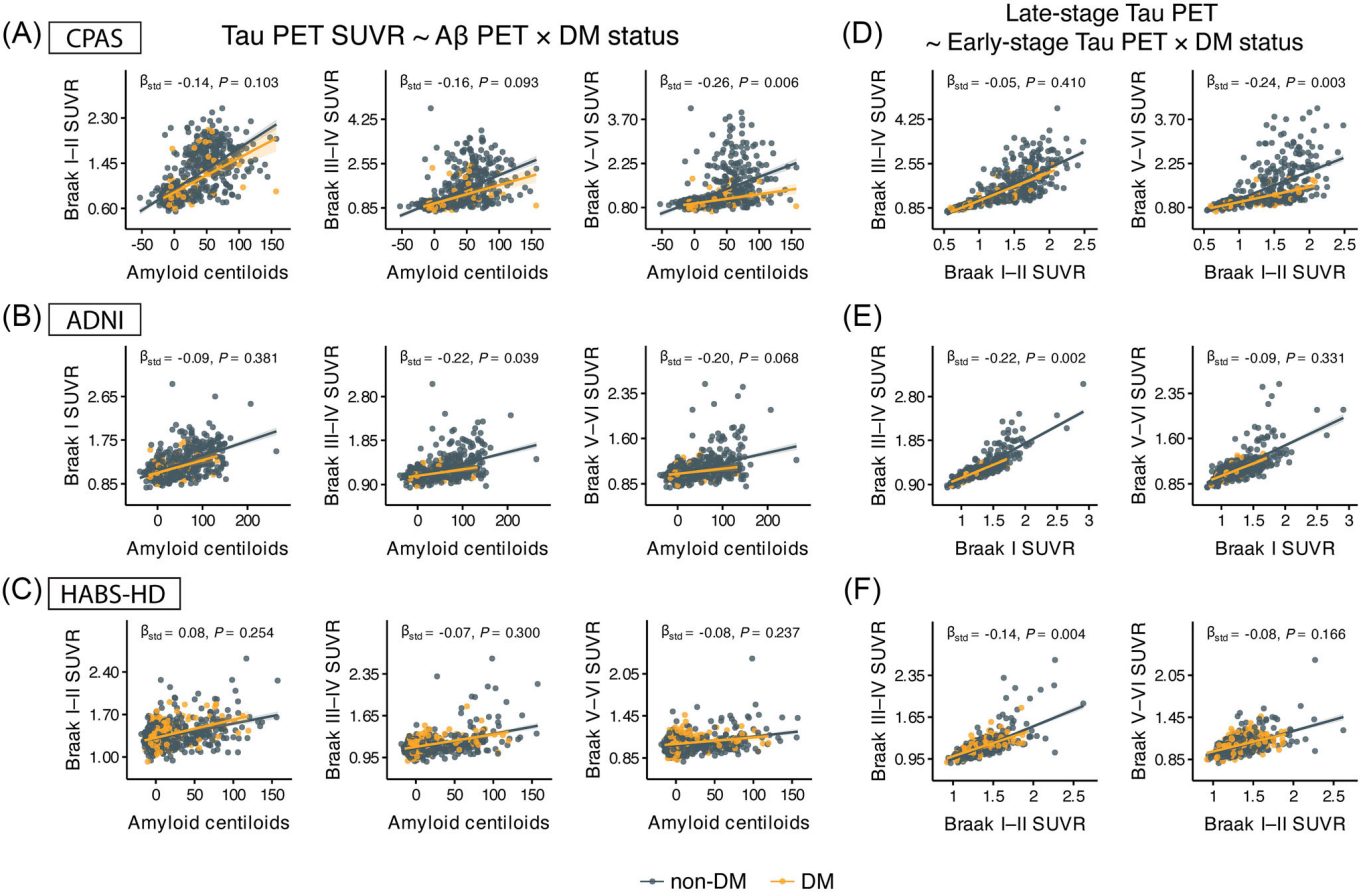
Diabetes mellitus modulates amyloid‐related tau accumulation and early–late tau association. A–C, Scatter plots illustrate the effect of DM status on the association between amyloid burden and regional tau accumulation across Braak regions (I–II, III–IV, and V–VI). D–F, Scatter plots illustrate the effect of DM status on the association between early‐stage tau accumulation (Braak I–II) and later stage tau accumulation (Braak III–IV and V–VI). Each point represents an individual participant. Solid lines show fitted linear regression models with shaded areas indicating 95% CIs. β values correspond to the interaction coefficients derived from regression models adjusted for age, sex, education, cognitive status, and *APOE* ε4 carrier status. *APOE*, apolipoprotein E; CI, confidence interval; DM, diabetes mellitus.

### 3.4 Diabetes attenuated tau propagation across Braak stages

We next examined whether the association between early‐ and late‐stage tau accumulation differed according to DM status by fitting linear mixed‐effects models including an interaction between DM and early‐stage tau (Braak I–II SUVR), adjusted for the same covariates. In CPAS, higher Braak I and II tau was less associated with Braak V and VI tau among participants with DM, as indicated by a significant DM × Braak I and II tau interaction (β = −0.24, 95% CI −0.40 to −0.08, *p* = 0.003; Figure 3D). In ADNI and HABS‐HD, DM attenuated the association between Braak I/I and II and Braak III and IV tau (ADNI: β = −0.22, 95% CI −0.35 to −0.08, *p* = 0.002; HABS‐HD: β = −0.14, 95% CI −0.23 to −0.04, *p* = 0.004; Figure 3E‐F).

### 3.5 Diabetes moderated longitudinal tau‐related cognitive decline

We then assessed whether the association between baseline AD pathology and longitudinal cognitive decline differed according to DM status in ADNI. Significant interactions were observed between diabetes and baseline tau accumulation in models predicting longitudinal changes in the MMSE (Braak I: β = 0.573, *p* = 0.011; Braak III–IV: β = 0.672, *p* = 0.032; Braak V–VI: β = 0.957, *p* = 0.006; Figure 4A), indicating that for a given level of baseline tau accumulation, participants with DM exhibited slower tau‐related cognitive decline.

**FIGURE 4:**
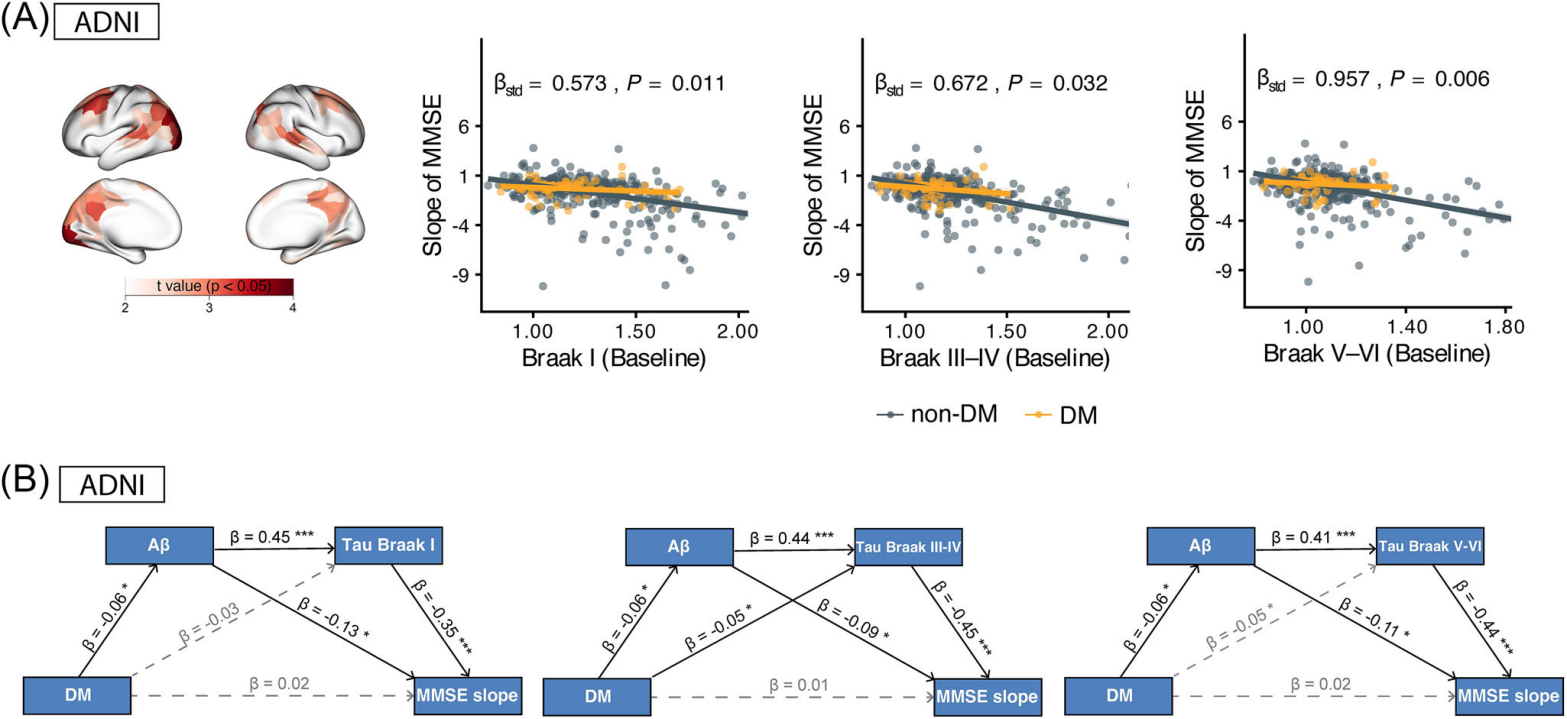
Interaction between baseline tau accumulation and diabetes mellitus status on longitudinal cognitive decline. A, Cortical surface maps show brain regions where baseline tau PET SUVR exhibits a significant interaction with DM status on the change rate of MMSE score. Positive *t* values (red) indicate that DM is associated with slower tau‐related MMSE decline compared to participants without DM. Scatter plots illustrate the effect of DM status on the association between baseline regional tau accumulation and the slope of MMSE change over time. B, Structural equation models (SEMs) depict the associations among DM status, baseline Aβ burden, baseline tau accumulation (Braak stage–specific), and longitudinal cognitive decline (MMSE slope). β values denote standardized path coefficients. Models were adjusted for age, sex, education, cognitive status, and *APOE* ε4 carrier status. Aβ, amyloid beta; *APOE*, apolipoprotein E; DM, diabetes mellitus; MMSE, Mini‐Mental State Examination; PET, positron emission tomography; SUVR, standardized uptake value ratio.

To further characterize the relationships among DM, AD pathology, and longitudinal cognitive change, structural equation modeling showed that DM was associated with lower Aβ burden (β = –0.06; 95% CI, −0.35 to −0.01, *p* = 0.03). Lower Aβ burden was, in turn, associated with reduced tau accumulation across all Braak regions; and both lower Aβ and tau burden were associated with a slower rate of MMSE decline (Figure 4B). Significant total indirect effects of DM on MMSE slope through Aβ and tau pathways were observed at Braak I (β = 0.08; 95% CI, 0.01 to 0.16; *p* = 0.048), Braak III and IV (β = 0.12; 95% CI, 0.04 to 0.22; *p* = 0.011), and Braak V and VI (β = 0.10; 95% CI, 0.02 to 0.18; *p* = 0.023).

### 3.6 Exploratory associations of antidiabetic medication use with AD‐related pathology

Finally, we exploratorily examined whether antidiabetic medication use was associated with AD‐related pathology in CPAS and ADNI (medication details were unavailable in HABS‐HD). Compared to participants without DM, metformin users showed lower tau accumulation prominently in late‐stage regions (Braak V–VI) in CPAS (*p* = 0.023, Figure 5A) and across all Braak regions in ADNI (all *p* < 0.05, Figure 5B). Among participants with diabetes not receiving metformin, tau accumulation differed from that of non‐diabetic participants in ADNI at Braak III and IV (*p* = 0.041), with weaker evidence at Braak I (*p* = 0.062), and in CPAS at Braak V and VI (*p* = 0.099). Stratified analyses by CU and CI indicated that these associations were primarily present among CI participants and were not observed among CU individuals (Figure S5 in supporting information).

**FIGURE 5:**
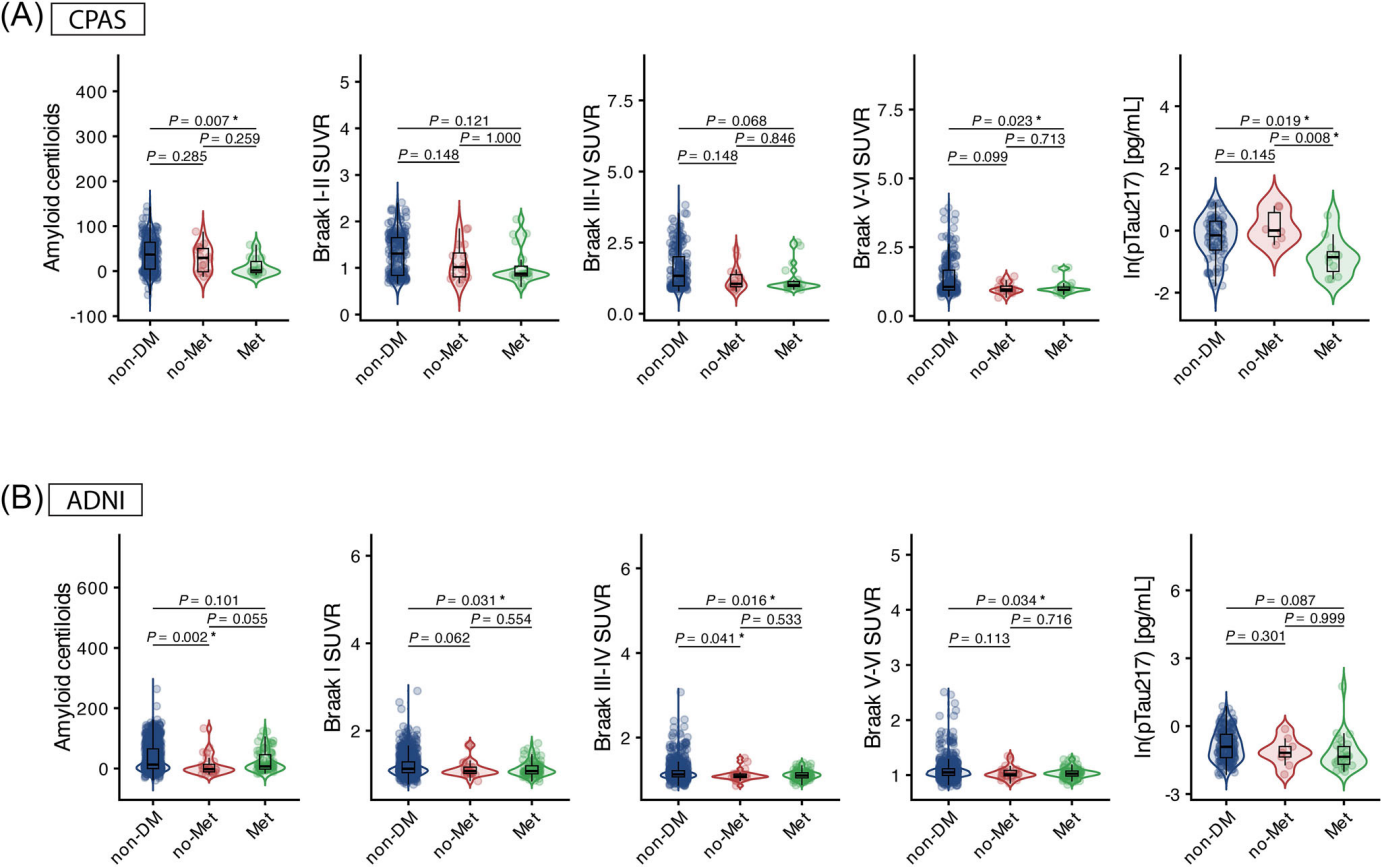
Association of metformin treatment with amyloid and tau burden. Comparison of amyloid burden, regional tau accumulation, and plasma p‐tau217 levels among individuals without diabetes mellitus (non‐DM), individuals with DM not taking metformin (no‐Met), and individuals with DM taking metformin (Met), in the CPAS (A) and ADNI (B) cohorts. Horizontal lines indicate group medians. *p* values were derived from pairwise comparisons adjusted for age, sex, education, cognitive status, and *APOE* ε4 carrier status. ADNI, Alzheimer's Disease Neuroimaging Initiative; *APOE*, apolipoprotein E; CPAS, Chinese Preclinical Alzheimer's Disease Study; DM, diabetes mellitus; p‐tau, phosphorylated tau.

## 4 DISCUSSION

In this multicenter study across three independent cohorts, we examined the associations of DM with Aβ and tau pathology using amyloid and tau PET, and plasma p‐tau217. Contrary to the hypothesis that DM exacerbates AD pathology, we found that DM was associated with lower Aβ burden and tau accumulation, particularly among CI participants. DM attenuated plasma p‐tau217‐ and amyloid‐related tau accumulation and weakened the association between early and later Braak stage tau accumulation. Longitudinally, DM also attenuated tau‐related cognitive decline, with slower decline observed at a given level of baseline tau accumulation. In exploratory analyses, metformin use among participants with DM was associated with lower tau accumulation. These findings suggest that DM, and potentially its treatment, may be associated with an altered relationship between AD biomarkers and cognitive progression.

We found that DM was associated with lower Aβ and tau burden, with these inverse associations being most pronounced among CI individuals. This finding is consistent with large‐scale neuropathological evidence. Autopsy studies have shown that individuals with DM do not exhibit increased global amyloid plaque or neurofibrillary tangle burden compared to non‐DM individuals, and in some studies, AD pathology is even less frequently observed in individuals with DM ^31^, ^32^, ^33^, ^34^, ^35^. However, the lower amyloid or tau burden in participants with DM should not be interpreted as evidence of neuroprotection. Instead, evidence suggests that DM is more strongly associated with cerebrovascular lesions and other non‐AD pathologies. Such vascular and metabolic brain injury may contribute to cognitive impairment in DM. Thus, the findings may reflect pathological substitution rather than protection against AD pathology. Consistent with this, amyloid PET studies report either no association or inverse associations between DM and fibrillar Aβ deposition ^17^, ^36^. However, in population‐based data, a diagnosis of diabetes ≈ 7 years prior to PET imaging has been linked to higher amyloid deposition ^15^, underscoring the heterogeneity and stage dependence of DM effects on AD pathology.

Our cohort‐level pattern supports this stage‐dependent interpretation. In CPAS and ADNI cohorts, which contain a higher proportion of CI individuals, DM was consistently associated with lower Aβ and tau burden, whereas in HABS‐HD, which is predominantly CU, we did not observe a comparable reduction in Aβ or tau; instead, DM was linked to higher late‐stage tau in CU participants. This stage‐dependent pattern is further supported by large‐scale biomarker evidence. In a meta‐analysis including > 5500 participants, DM was associated with lower odds of abnormal Aβ biomarkers in CI individuals, while higher odds of abnormal neurodegeneration were observed primarily among CU participants ^18^. However, individuals with DM who survived and were sufficiently healthy to enroll and undergo PET imaging may represent a selected subgroup. Other individuals with more severe metabolic or vascular disease, or a greater combined burden of vascular and AD pathology, may have died earlier or been less likely to enter or remain in these cohorts. Therefore, these findings should not be interpreted as evidence that DM is protective at later disease stages.

Beyond the lower overall Aβ and tau burden, diabetes was associated with attenuated Aβ‐related tau and plasma p‐tau217–related tau. This result suggests that tau accumulation in DM may be less linked to amyloid‐related pathways than in non‐diabetic individuals. Existing evidence shows that DM is strongly associated with cerebrovascular injury, metabolic dysregulation, and chronic inflammation, all of which can independently contribute to neurodegeneration and cognitive impairment ^31^, ^33^, ^37^, ^38^. Therefore, non‐amyloidogenic cascades may contribute more prominently to the pathology related to cognitive decline in DM individuals, thereby reducing the burden of classical amyloid–tau pathology required at the symptomatic stage.

Notably, our exploratory analyses did not identify evidence that diabetes modified the association between plasma GFAP (i.e., a marker of astrocytic activation) and Aβ or tau burden. It provides limited support for astrocytic activation (as indexed by GFAP) as the main contributor; however, this finding does not exclude the contribution of inflammation via other pathways. Another possible explanation for the lower Aβ‐ and p‐tau217–related tau accumulation observed in DM may be disease management. In our study, lower Aβ burden and tau accumulation were observed among metformin‐treated participants with DM than among participants without DM. Metformin use was associated with a lower tau accumulation, with weaker evidence for Aβ. This result is supported by preclinical studies suggesting that metformin modulates adenosine monophosphate activated protein kinase (AMPK) signaling and enhances protein phosphatase‐2A (PP2A) activity, thereby limiting tau phosphorylation ^39^, ^40^, promoting the clearance of pathological protein aggregates and limiting the spatial propagation of tau ^41^, ^42^. Although earlier animal studies raised concerns about potential amyloidogenic effects of metformin via beta‐secretase 1 upregulation ^43^, other experimental data, together with our findings, suggest a net neuroprotective effect and reduction of Aβ ^41^, ^44^. In addition to metformin, other glucose‐lowering therapies including glucagon‐like peptide‐1 receptor agonists and insulin, have been shown to exert neuroprotective effects that extend beyond glycemic control ^45^, ^46^, ^47^, ^48^, ^49^, ^50^. The direct comparisons between metformin users and non‐users with DM showed no consistent differences in Aβ or tau PET measures. The observed patterns may therefore relate more broadly to diabetes management, including glycemic control and the use of other glucose‐lowering medications.

Although diabetes is a risk factor for dementia, our longitudinal analyses in ADNI did not show faster cognitive decline among participants with diabetes. Instead, DM moderated the association between baseline tau accumulation and subsequent MMSE change, such that higher tau was less strongly coupled to cognitive decline in individuals with diabetes. Together with our cross‐sectional findings of lower Aβ and tau accumulation in diabetes in the more CI cohorts (i.e., CPAS and ADNI), these results support the notion that diabetes does not necessarily accelerate tau‐driven cognitive decline once substantial AD pathology is already present. Instead, DM may increase vulnerability to cognitive impairment through non‐AD mechanisms; or DM management may additionally modulate the AD‐related pathology and attenuate the rate of cognitive decline. This result aligns with a recent finding that DM was not associated with faster longitudinal brain Aβ deposition ^51^.

A key strength of this study is that it represents, to our knowledge, the largest multi‐cohort study to date examining the association between DM and AD‐related neuropathology in which all participants underwent both amyloid and tau PET (*N* = 2784), and with complementary plasma p‐tau217 measures available in a subset of participants. By analyzing data across three multicenter cohorts, we provide mechanistic insight into the interplay between DM and AD pathology. Several limitations should be acknowledged. First, detailed information on antidiabetic treatment exposure (e.g., duration, dosage), and differentiation between monotherapy and polypharmacy, was not available across cohorts; therefore, future studies should systematically investigate the effects of diabetes management on AD pathology. Second, longitudinal imaging data were not consistently available across cohorts, which constrained the assessment of long‐term Aβ and tau trajectories. Third, we did not differentiate type 1 and type 2 diabetes, precluding diabetes subtype‐specific analyses; although type 2 diabetes is much more frequent than type 1 in older people. Fourth, the duration of cognitive impairment and time since diagnosis were not consistently available across cohorts. Although the associations of DM with lower amyloid and tau burden remained significant after adjustment for baseline MMSE, residual confounding by disease duration or other aspects of clinical stage cannot be excluded.

## 5 CONCLUSION

This multicohort study shows that diabetes mellitus is associated with lower tau pathology and an attenuated amyloid–tau association, reduced propagation of tau pathology, and slower tau‐related cognitive decline. Metformin use among individuals with DM was associated with lower tau accumulation. These findings suggest that diabetes, and potentially its management, may affect AD pathology, highlighting the need to develop diabetes‐specific management strategies for individuals with or at risk of dementia.

## AUTHOR CONTRIBUTIONS

M.W.: conceptualization, methodology, data analysis, writing – original draft, writing – review and editing. Y.L.: conceptualization, methodology, data analysis, writing – review and editing. X.X.: methodology, data analysis, writing – review and editing; X.M.: conceptualization, data acquisition, writing – review and editing; H.L.: data acquisition, writing – review and editing; K.H.: data acquisition, writing – review and editing; Q.H.: methodology, writing – review and editing. J.Z.: methodology, writing – review and editing; S.K.: methodology, writing – review and editing; Y.G.: methodology, resources, funding acquisition, writing – review and editing; S.B.: resources, writing – review and editing; B.L.: data acquisition, resources, writing – review and editing; Q.G.: data acquisition, resources, writing – review and editing; B.Y.: conceptualization, supervision, resources, funding acquisition, writing – review and editing; F.X.: conceptualization, study design, methodology, supervision, resources, funding acquisition, writing – review and editing.

## CONFLICT OF INTEREST STATEMENT

All authors declare that they have no conflicts of interest to disclose. Author disclosures are available in the Supporting Information.

## CONSENT STATEMENT

All human subjects provided informed consent.

## Supporting information



Supporting Information

Supporting Information

## Data Availability

Data supporting the findings of this study are available from the ADNI database and HABS‐HD database upon registration and compliance with the data use agreement. Data from CPAS cannot be publicly shared due to Chinese data protection rules. Requests for data access can be sent to the corresponding author (F.X., Fangxie@fudan.edu.cn) as a formal proposal specifying the purpose of the data request.
