# Supplementary material for: Diabetes mellitus is associated with altered Alzheimer's disease pathology
Source: Alzheimers Dement. 2026 Nov 10;22(11):e72002. doi: 10.1002/alz.72002 (PMC13751832; doi:10.1002/alz.72002)
Supplement: Supplementary file 2 [file ALZ-22-e72002-s001.docx]

**Supplementary Material**

**Diabetes mellitus is associated with altered Alzheimer’s disease pathology**

Min Wu, Ying Luan, Xiaowen Xu, Xiaoxie Mao, Huiying Lin, Kun He, Qi Huang, Jie Zhang, Sheng Kuang, Yihui Guan, for the Alzheimer’s Disease Neuroimaging Initiative, the Health and Aging Brain Study (HABS-HD) Team, Sid O'Bryant, Binyin Li, Qihao Guo, Fang Xie, Benyan Luo

**Supplementary Table S1.** Participant characteristics stratified by cognitive status and diabetes status

| **CPAS (N = 734)** | | | | | | | |  |
| --- | --- | --- | --- | --- | --- | --- | --- | --- |
| Characteristic | Total (N = 734) | CU, non-DM (n = 207) | CU, DM (n = 40) | P value | CI, non-DM (n = 412) | CI, DM (n = 75) | P value | |
| Age, mean (SD), y | 68.1 (8.2) | 65.3 (8.4) | 65.8 (6.7) | .74 | 69.1 (8.0) | 71.4 (6.8) | .02 | |
| Sex, No. (%) |  |  |  | .01 |  |  | .38 | |
| Female | 453 (61.7) | 140 (67.6) | 18 (45.0) |  | 253 (61.4) | 42 (56.0) |  | |
| Male | 281 (38.3) | 67 (32.4) | 22 (55.0) |  | 159 (38.6) | 33 (44.0) |  | |
| Education, mean (SD), y | 11.6 (3.8) | 13.1 (3.3) | 12.7 (3.5) | .47 | 10.6 (3.8) | 11.9 (4.2) | .01 | |
| APOE e4 carriers, No. (%) | 279 (38.0) | 42 (20.3) | 10 (25.0) | .50 | 203 (49.3) | 24 (32.0) | .01 | |
| Amyloid-beta positive, No. (%) | 370 (50.4) | 41 (19.8) | 5 (12.5) | .28 | 286 (69.4) | 38 (50.7) | <.001 | |
| Baseline Biomarkers |  |  |  |  |  |  |  | |
| Plasma p-tau217, mean (SD), pg/mL | 0.7 (0.6) | 0.3 (0.2) | 0.4 (0.2) | .74 | 0.9 (0.6) | 0.7 (0.6) | .18 | |
| Plasma GFAP, mean (SD), pg/mL | 101.4 (88.5) | 55.2 (28.1) | 62.5 (50.5) | .35 | 128.4 (102.6) | 90.7 (59.5) | .03 | |
| Amyloid PET, mean (SD), Centiloids | 24.7 (36.4) | 2.9 (18.0) | 1.2 (15.4) | .6 | 38.6 (38.5) | 21.8 (33.8) | <.001 | |
| Tau PET (Braak I-II), mean (SD), SUVR | 1.1 (0.4) | 0.9 (0.2) | 0.8 (0.1) | .26 | 1.3 (0.4) | 1.1 (0.4) | <.001 | |
| Tau PET (Braak III-IV), mean (SD), SUVR | 1.3 (0.6) | 1.0 (0.1) | 1.0 (0.1) | .26 | 1.6 (0.7) | 1.2 (0.5) | <.001 | |
| Tau PET (Braak V-VI), mean (SD), SUVR | 1.2 (0.5) | 0.9 (0.1) | 0.9 (0.1) | .01 | 1.4 (0.7) | 1.0 (0.2) | <.001 | |
| MMSE score, mean (SD) | 24.1 (5.4) | 28.1 (1.6) | 28.0 (1.6) | .65 | 21.8 (5.6) | 24.0 (5.0) | <.001 | |
|  |  |  |  |  |  |  |  | |
| **ADNI (N = 835)** | | | | | | | |  |
| Characteristic | All (N = 835) | CU, non-DM (n = 406) | CU, DM (n = 56) | P value | CI, non-DM (n = 320) | CI, DM (n = 53) | P value | |
| Age, mean (SD), y | 73.7 (7.9) | 72.7 (7.7) | 73.0 (6.4) | .78 | 75.1 (8.1) | 73.9 (8.6) | .35 | |
| Sex, No. (%) |  |  |  | .25 |  |  | .66 | |
| Female | 430 (51.5) | 243 (59.9) | 29 (51.8) |  | 137 (42.8) | 21 (39.6) |  | |
| Male | 405 (48.5) | 163 (40.1) | 27 (48.2) |  | 183 (57.2) | 32 (60.4) |  | |
| Education, mean (SD), y | 16.4 (2.5) | 16.8 (2.3) | 16.3 (2.4) | .14 | 16.1 (2.5) | 15.7 (2.9) | .36 | |
| APOE e4 carriers, No. (%) | 315 (37.7) | 132 (32.5) | 17 (30.4) | .75 | 152 (47.5) | 14 (26.4) | <.001 | |
| Amyloid-beta positive, No. (%) | 341 (40.8) | 128 (31.5) | 9 (16.1) | .02 | 181 (56.6) | 23 (43.4) | .07 | |
| Baseline Biomarkers |  |  |  |  |  |  |  | |
| Plasma p-tau217, mean (SD), pg/mL | 0.5 (0.5) | 0.5 (0.4) | 0.3 (0.1) | <.001 | 0.6 (0.5) | 0.8 (1.5) | .33 | |
| Plasma GFAP, mean (SD), pg/mL | 182.9 (92.9) | 185.8 (90.4) | 139.9 (59.0) | .04 | 198.1 (104.3) | 149.0 (73.5) | .09 | |
| Amyloid PET, mean (SD), Centiloids | 31.7 (44.7) | 20.8 (34.7) | 9.9 (21.2) | .02 | 49.6 (52.6) | 30.2 (42.9) | .01 | |
| Tau PET (Braak I), mean (SD), SUVR | 1.2 (0.2) | 1.1 (0.1) | 1.1 (0.1) | .23 | 1.3 (0.3) | 1.2 (0.2) | <.001 | |
| Tau PET (Braak III-IV), mean (SD), SUVR | 1.2 (0.2) | 1.1 (0.1) | 1.1 (0.1) | .23 | 1.3 (0.3) | 1.1 (0.1) | <.001 | |
| Tau PET (Braak V-VI), mean (SD), SUVR | 1.1 (0.2) | 1.0 (0.1) | 1.0 (0.1) | .18 | 1.1 (0.2) | 1.0 (0.1) | .01 | |
| MMSE score, mean (SD) | 28.0 (2.7) | 29.1 (1.2) | 29.2 (0.9) | .54 | 26.6 (3.5) | 27.0 (2.8) | .35 | |
| Longitudinal Data |  |  |  |  |  |  |  | |
| Participants with follow-up, No. (%) | 625 (74.9) | 308 (75.9) | 41 (73.2) | .67 | 237 (74.1) | 39 (73.6) | .94 | |
| Follow-up duration, mean (SD), y | 3.5 (1.7) | 3.9 (1.6) | 3.6 (1.7) | .25 | 2.9 (1.7) | 3.0 (1.6) | .70 | |
|  |  |  |  |  |  |  |  | |
| **HABS-HD (N = 1215)** | | | | | | | |  |
| Characteristic | All (N = 1215) | CU, non-DM (n = 655) | CU, DM (n = 221) | P value | CI, non-DM (n = 228) | CI, DM (n = 111) | P value | |
| Age, mean (SD), y | 65.7 (8.5) | 65.4 (8.3) | 65.4 (8.1) | .96 | 65.9 (9.3) | 66.9 (9.1) | .36 | |
| Sex, No. (%) |  |  |  | .19 |  |  | .39 | |
| Female | 735 (60.5) | 435 (66.4) | 136 (61.5) |  | 114 (50.0) | 50 (45.0) |  | |
| Male | 480 (39.5) | 220 (33.6) | 85 (38.5) |  | 114 (50.0) | 61 (55.0) |  | |
| Education, mean (SD), y | 14.2 (3.4) | 15.0 (3.0) | 13.1 (4.0) | <.001 | 13.8 (3.5) | 12.8 (3.6) | .01 | |
| APOE e4 carriers, No. (%) | 371 (30.5) | 196 (29.9) | 54 (24.4) | .12 | 84 (36.8) | 37 (33.3) | .53 | |
| Amyloid-beta positive, No. (%) | 222 (18.3) | 106 (16.2) | 27 (12.2) | .16 | 65 (28.5) | 24 (21.6) | .18 | |
| Baseline Biomarkers |  |  |  |  |  |  |  | |
| Plasma p-tau217, mean (SD), pg/mL | 0.4 (0.4) | 0.4 (0.2) | 0.4 (0.3) | .14 | 0.7 (0.7) | 0.4 (0.3) | .12 | |
| Plasma GFAP, mean (SD), pg/mL | 116.4 (68.3) | 118.6 (66.1) | 115.2 (66.5) | .71 | 116.3 (73.0) | 108.8 (74.7) | .60 | |
| Amyloid PET, mean (SD), Centiloids | 10.3 (25.4) | 8.6 (23.9) | 6.3 (16.8) | .19 | 17.1 (33.0) | 13.9 (27.1) | .38 | |
| Tau PET (Braak I-II), mean (SD), SUVR | 1.3 (0.2) | 1.3 (0.1) | 1.3 (0.1) | .35 | 1.4 (0.2) | 1.4 (0.2) | .81 | |
| Tau PET (Braak III-IV), mean (SD), SUVR | 1.1 (0.1) | 1.1 (0.1) | 1.1 (0.1) | <.001 | 1.2 (0.2) | 1.2 (0.1) | .53 | |
| Tau PET (Braak V-VI), mean (SD), SUVR | 1.0 (0.1) | 1.0 (0.1) | 1.1 (0.1) | <.001 | 1.1 (0.2) | 1.1 (0.1) | .50 | |
| MMSE score, mean (SD) | 27.8 (2.5) | 28.6 (1.6) | 27.8 (2.0) | <.001 | 26.4 (3.4) | 26.1 (3.0) | .40 | |

**Supplementary Figure S1. Association of diabetes mellitus with amyloid and tau burden stratified by cognitive status.** (A–C). Cortical surface maps show amyloid and tau PET SUVR in DM and non-DM participants, stratified by cognitive status (CU and CI), across CPAS (A), ADNI (B), and HABS-HD (C). (D–F). Box plots compare DM versus non-DM groups for amyloid burden, tau burden, plasma p-tau217, and MMSE within CU and CI groups. Effects are reported as standardized regression coefficients (negative β values indicate lower amyloid or tau burden or lower MMSE scores in the DM group), with 95% CIs. Models were adjusted for age, sex, education, and APOE ε4 carrier status.

**Supplementary Figure S2. Interaction between diabetes mellitus and plasma p-tau217 on tau burden stratified by cognitive status.** The effect of DM on the association between plasma p-tau217 levels and regional tau accumulation across Braak stages I–II, III–IV, and V–VI, separately for CU and CI participants in the CPAS (A), ADNI (B), and HABS-HD (C) cohorts. β values represent the DM × p-tau217 interaction.

**Supplementary Figure S3. Diabetes mellitus modifies amyloid-related tau burden and early-late tau association.** (A–C). The effect of DM status on the association between amyloid burden and regional tau accumulation across Braak regions (I–II, III–IV, and V–VI), shown separately for CU and CI participants in the CPAS, ADNI, and HABS-HD cohorts. (D–F). The effect of DM status on the association between early-stage tau burden (Braak I–II SUVR) and later-stage tau burden (Braak III–IV and V–VI SUVR). β values represent interaction coefficients derived from regression models adjusted for age, sex, education, and APOE ε4 carrier status.

**Supplementary Figure S4. Interaction between diabetes mellitus status and GFAP on amyloid and tau burden.** (A–C). Scatter plots depict the effect of DM status on the association between plasma GFAP and amyloid burden or regional tau accumulation across Braak regions (I–II, III–IV and V–VI). Each point represents an individual participant. Solid lines show linear regression fits and shaded areas indicate 95% CIs. β values represent the interaction coefficients derived from regression models adjusted for age, sex, education, cognitive status and APOE ε4 carrier status.

**Supplementary Figure S5.** **Association of metformin treatment with amyloid and tau burden stratified by cognitive status.** Comparison of amyloid burden, regional tau accumulation, and plasma p-tau217 levels among non-DM participants, DM participants not taking metformin (no-Met), and DM participants taking metformin (Met), in the CPAS (A) and ADNI (B) cohorts, separately for the CU and CI groups. Horizontal lines indicate group medians. P values were derived from pairwise comparisons adjusted for age, sex, education and APOE ε4 carrier status.

**Supplementary Figure S6. The effects of APOE ε4 carrier status on the Aβ and tau burden, plasma p-tau217 and MMSE.** Comparison of amyloid burden, tau burden, plasma p-tau217, and MMSE between the APOE ε4 and APOE non-ε4 carriers within non-DM and DM groups. Effects are reported as standardized regression coefficients, with 95% CIs. Models were adjusted for age, sex and education.

**Supplementary Figure S7. Association of diabetes mellitus with amyloid and tau burden stratified by amyloid status.** Similar to Figure 1, but for the comparison of amyloid and tau burden between DM and non-DM in Aβ+ and Aβ- individuals. The amyloid positivity cutoff was defined by Gaussian mixture modeling.

**Supplementary Figure S8.** Association of diabetes mellitus with amyloid and tau burden stratified by cognitive status after additional adjustment for white matter hyperintensity volume. (A) Log-transformed white matter hyperintensity (WMH) volume in participants with and without diabetes mellitus (DM), stratified by cognitive status, in ADNI and HABS-HD. (B-C). DM effects on amyloid and tau burden after further adjustment of WMH volume.
